# Driving evaluation methods for able-bodied persons and individuals with lower extremity disabilities: a review of assessment modalities

**DOI:** 10.6061/clinics/2015(09)08

**Published:** 2015-09

**Authors:** Julia Maria D'Andréa Greve, Luciana Santos, Angelica Castilho Alonso, Denise G Tate

**Affiliations:** I Faculdade de Medicina da Universidade de São Paulo, Department of Orthopedics and Traumatology, São Paulo/SP, Brazil; II University of Michigan Medical School, Department of Physical Medicine and Rehabilitation, Ann Arbor, MI, USA

**Keywords:** Automobile Driving, Amputation, Spinal Cord Injury, Driving Assessment

## Abstract

Assessing the driving abilities of individuals with disabilities is often a very challenging task because each medical condition is accompanied by physical impairments and because relative individual functional performance may vary depending on personal characteristics.

We identified existing driving evaluation modalities for able-bodied and lower extremity-impaired subjects (spinal cord injury patients and amputees) and evaluated the potential relationships between driving performance and the motor component of driving.

An extensive scoping review of the literature was conducted to identify driving assessment tools that are currently used for able-bodied individuals and for those with spinal cord injury or lower extremity amputation. The literature search focused on the assessment of the motor component of driving. References were electronically obtained via Medline from the PubMed, Ovid, Web of Science and Google Scholar databases.

This article compares the current assessments of driving performance for those with lower extremity impairments with the assessments used for able-bodied persons. Very few articles were found concerning “Lower Extremity Disabilities,” thus confirming the need for further studies that can provide evidence and guidance for such assessments in the future. Little is known about the motor component of driving and its association with the other driving domains, such as vision and cognition. The available research demonstrates the need for a more evidenced-based understanding of how to best evaluate persons with lower extremity impairment.

## INTRODUCTION

The need to evaluate drivers′ ability to safely operate a motor vehicle has been increasing, especially due to the aging of the global population and the use of sophisticated technologies in newer vehicles 1-3. The aging process is accompanied by many health conditions that can affect one's ability to drive, including amputations and spinal cord injury (SCI) due to medical or traumatic events 4-6

There are numerous methods of assessing driving abilities in the general population 3,7. While some researchers have been working towards the development of a battery assessment to predict crash risk, others have been focused on identifying an easily administered instrument that can identify those who are fit to drive**.** The existing driving assessment protocols rely on physical, visual and cognitive capabilities, all of which are essential skills for the safe operation of a motor vehicle 8-13.

The literature is quite comprehensive regarding driving safety and assessment tools for certain unique groups and for those with specific clinical conditions such as Parkinson's disease, dementia, traumatic brain injury and stroke 14-16. Several measures and models are currently being used in driving assessments, but very few references are found regarding the ability of these assessment protocols to determine driving performance among individuals with lower extremity functional motor impairment that precludes the use of the lower legs and feet while operating a vehicle 17.

Notwithstanding the severity of their disorder, every individual should undergo a thorough assessment of their driving capabilities to provide them with accurate feedback and to better guide them concerning their driving options 1,14-16,. There is a lack of information regarding the driving abilities of both SCI patients and amputees. There are no clear guidelines or consensus recommendations to assist health care professionals in developing driving evaluation protocols for these patients, and in the case of amputees, there is no clear evidence defining the appropriate assessment measures or even adaptive aids or the car modifications required to compensate for the lack of motor and sensory function 19,20.

The lack of information in this area is further compromised by the fact that driving is a multifaceted task that involves the integration of different body systems – motor, visual, cognitive, and behavioral – and that requires complex protocols for evaluation 7,21. Impairments in any these systems can affect a driver's performance behind the wheel. Although some researchers are trying to integrate tests and develop tools to assess the impaired individual as a whole, the current driving evaluation protocols rely on the application of several isolated tests for each system. Thus, appraising an individual's driving ability is quite challenging, especially for those with certain medical conditions in which the manifestations of the disease and its sequelae vary from person to person 5,22.

Even though it has been difficult to predict, a significant portion of the literature describes risk factors leading to vehicle crashes. Because all drivers (including able-bodied individuals and those with specific medical conditions or disabilities) are exposed to uncontrolled external conditions in the environment, such as poor road conditions, heavy traffic, and distracting events, all of which can lead to accidents, the role of a driving assessment is to evaluate the driver's skills within the context of these difficult conditions as accurately as possible. The identification of risk factors in such evaluations is the key to preventing involvement in motor vehicle accidents. In reviewing the literature, authors have taken these factors into consideration.

In Brazil, all disabled persons must be evaluated by a medical team prior to obtaining their driver's license to determine which adaptations are recommended. Subsequently, they must submit to a practical driving test. In USA, the procedure is very similar, but the process of performing the evaluation and testing varies according to the laws of each state. Many rehabilitation centers in developed countries provide adaptation and training prior to the practical evaluation.

This paper is divided into two distinct parts. First, we describe the current driving assessments for the general population and for individuals with lower extremity impairment due to SCI or amputation. Second, we discuss the potential relationships between the motor dimension of the driving assessment and driving ability, with a focus on the physical evaluation and its respective tools/tests as described by each selected study. Third, we offer recommendations based on the literature reviewed. This article focuses on the motor component of driving because this is the most critical element in individuals with lower extremity impairments. In general, neither cognition nor vision is affected in individuals with lower extremity impairments.

## METHODS

**Figure 1:**
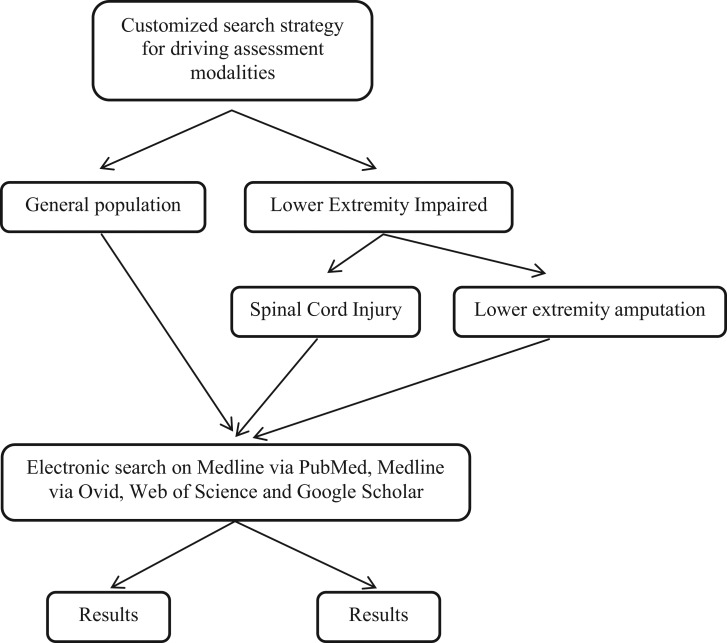
Detailed search scheme used to retrieve manuscripts related to driving assessment for the general population and for lower extremity-impaired individuals.

An extensive scoping review of the literature was conducted to identify driving assessment tools used for the general population and for people with lower extremity impairment. In our selection of those with lower extremity impairment, we focused our review on individuals with SCI, neurologically classified paraplegia, and those with amputation of a lower limb or below the knee. References were electronically obtained via Medline from the PubMed, Ovid, Scopus, Web of Science and Google Scholar databases from December 2014 to March 2015. We excluded the more technical databases such as Scopus, as a preliminary search returned substantial engineering content. To be as comprehensive as possible, no time or language limitations were applied to our search (Figure 1).

Search strategies, including appropriate keywords and controlled terms related to two distinct searches, were customized for these databases. The first search retrieved articles that discussed current assessment modalities used to evaluate driving for the general population. This search was considered to be an important step to gain a basic understanding of the contributions to the literature in this area and to be able to contrast these results with those of the second search. A more specific search was then performed on three major concept areas: persons with SCI (spinal cord injury*, “spinal cord injuries”[mesh], etc.), persons with lower extremity amputation [ex. (amput* OR “amputees”)(mesh)] AND (lower OR leg OR tibia* OR foot OR feet OR toe* OR knee OR hip), and driver assessment [(ex. (driver* OR “automobile driving”[mesh] OR traffic* OR “fitness to drive”)] AND (assess* OR tool* OR protocol OR test*). Further details of these searches are available upon request. All searches were performed with the assistance of a medical librarian.

While the search strategies were initially designed to target motor assessment, the narrow set of results from each database led to the decision to broaden the searches to all forms of driving assessments in these populations to return sufficiently comprehensive data. Due to inconsistent indexing practices, ambiguous terminology and a clear scarcity of research on the lower extremity-impaired population, additional searches and search strategies were performed using all databases and the open web to identify driving assessment tools in other populations with chronic conditions or disabilities. The references retrieved from this search were manually inspected, and reference tracking was used to identify additional relevant content. These results formed the basis of the literature review presented here.

Among the pool of available articles, we gathered those which included motor assessment in their protocol, regardless of study type and outcomes. Within each selected article, we focused on the motor part of the evaluation, especially the methods of assessment, the study outcomes when available and other topics of interest that could contribute to the development of recommendations for future studies.

## RESULTS

### Overall Findings

**Figure 2:**
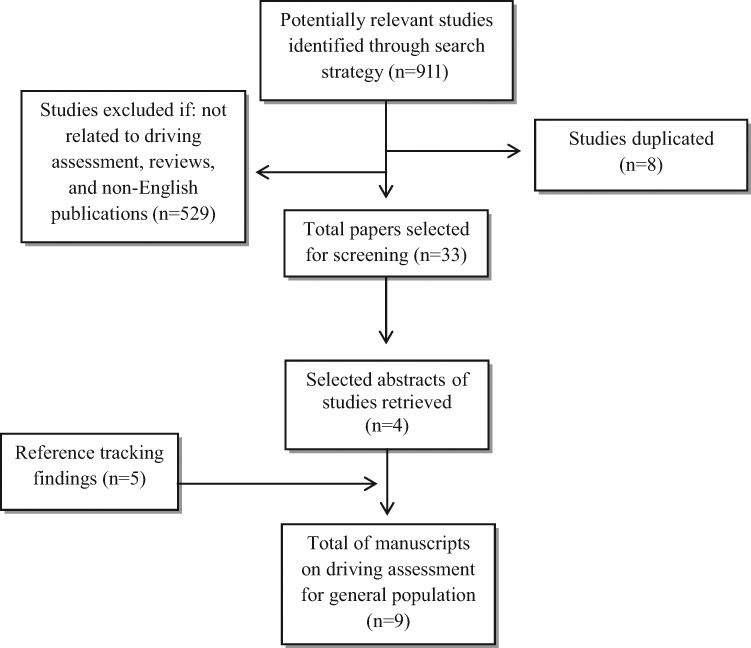
Flowchart displaying screening process and search results of driving assessment for general population.

**Figure 3:**
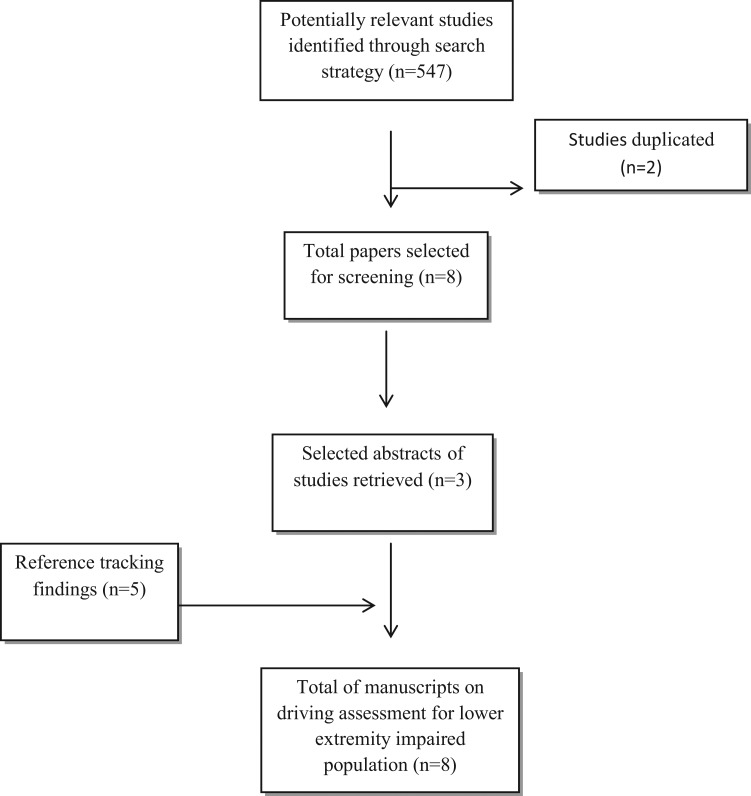
Flowchart displaying detailed screening process and results of the search for driving assessment modalities for lower extremity impaired population.

Based on an extensive scoping review, we identified a total of 911 potentially relevant studies related to driving assessment for the general population and 547 studies related to driving assessment for those with lower extremity impairment due to any condition or diagnosis. See Figures 2 and 3.

We found only nine studies 1-3,7,8,21, under the general population search that described motor performance evaluations as components of driving assessment protocols. Most of these papers discussed the performance of older drivers and described specific motor assessment tests such as range of motion, rapid pace walk, strength, foot tap, arm reach, timed up and go and one leg stance. Neck range of motion was included in all of the studies, and hand grip was included in at least three studies. Two studies focused on the relationship between physical assessment and crash involvement, and three others investigated the likelihood to include motor assessment in a potential driving assessment model (Table 1).

Our search identified eight studies 17,19,20, related to driving assessment for persons with SCI or lower limb amputation. Of those studies, three were specifically designed to assess SCI subjects, and two focused on amputees. Three manuscripts discussed driving assessments for a general sample of individuals with physical disabilities, including SCI or amputation. Five of the studies assessed driving performance using a driving simulator, and one using a road test. Two papers studied pedal reaction time (RT) among amputees. One of these studies explored the relationship between RT and cognitive performance, the other study assessed the influence of different techniques of pedal operation on RT (Table 2). None of the examined papers concerning driving performance after SCI clearly explained how to perform an assessment of the motor component of driving.

### Assessing driving ability in the general population

Among the able-bodied population, the literature lists several factors associated with poor driving performance. Vision, cognition and motor abilities play separate roles in the capability to drive safely among able-bodied individuals 25,31. All of the reviewed driving assessment protocols were developed using older drivers due to the well-known factors associated with aging and physical health decline. Antin et al. 2 designed a protocol composed of three dimensions related to driving (perception, physical ability and visual-cognitive-ability) that accurately separates older drivers from older non-drivers based on their degree of functional impairment. These researchers encouraged retesting the model with a larger and more representative sample to validate the proposed protocol.

An affordable and comprehensive assessment battery consisting of a set of instruments that represents all of the dimensions of driving has been successfully developed for elderly drivers. However, protocols such as this battery must be modified for use in lower extremity-impaired individuals. The reasons for these modifications include 1) the use of compensatory motor skills when driving due to physical impairment and 2) possible high emotional distress associated with having to drive with limited physical function 23.

For example, the CanDRIVE study, which was developed in Canada, provides an outpatient screening protocol to help clinicians identify older drivers who may be unsuited to operate a motor vehicle or who require a comprehensive driving assessment to establish driving safety. In addition to including sensory, physical and cognitive assessment modalities, the system appraises psychosocial factors, driving habits/behaviors, health status, and on-road test performance 7.

In 2010, the American Medical Association proposed guidelines 32 to be used in-office to appraise people's overall fitness to drive; these guidelines were referred to as the Assessment of Driving-Related Skills (ADReS). Two of the selected papers examined the usefulness of this clinical tool, but they did not show evidence of the ability of ADReS to predict car accidents, explaining that its accuracy is not sufficiently high to warrant its use as a screening test 8,33.

In an attempt to develop a measure predictive of driving performance, Stav and colleagues 25 showed that all three driving task domains (vision, motor and cognition) should be represented by at least one test in a predictive driving protocol. Thus, considering the complexity of the driving task, Wood et al. 3 selected a set of tests to optimally predict safe driving. Their final predictor battery included a selection of tools representing various abilities that are needed to drive safely while reflecting the multifaceted nature of driving 3. Similarly, Ball et al. 21 used a full set of tests (assessing vision, motor and cognition) that predicted future at-fault motor vehicle collision (MVC). However, in their sample, the cognitive tests alone were the most predictive.

Only one study did not include all of the previously mentioned domains of driving assessment; instead, this study focused on the relationship of sensorimotor function to postural control and driving performance. This was the only study that attempted to clarify the association of sensorimotor risk factors with unsafe driving. The authors also reported several measures associated with driving safety. As discussed in their report, these findings could be used to develop strategies to improve people's overall fitness to drive via physical interventions designed for driver training and education 1.

### Assessing driving among those with lower extremity impairment

There is no clear evidence-based research or recommendation regarding how to properly assess fitness to drive among people with lower extremity impairment. The multidimensional task of driving an automobile is significantly more complex for those with lower extremity impairment than for the able-bodied population. Driving assessments must consider the need for car adaptations, the use of hand controls and prostheses and the new learning of motor and cognitive skills, all of which are required for safe driving by individuals with lower extremity impairment.

From a general physical disability perspective, the only study that attempted to validate criteria for assessing functional fitness to drive was conducted in the United Kingdom using a device termed the static assessment rig (SAR). The SAR includes a set of tests that evaluate steering force (torque), braking force and RT. Despite the researchers' efforts to identify cutoff values representing levels of driving performance, they found that the SAR could not be used alone as a screening tool but that it could assist in the determination of whether adaptation may be needed 17.

Several of the reviewed papers reported the use of a driving simulator to evaluate driving performance. All of these studies used similar outcome measures, including steering torque, braking force and RT. A study conducted by Lings et al. found that the main difference in RT between controls and SCI subjects occurred during movement time (the interval between the release of the accelerator and the operation of the brake). This study also found that greater neurological injury was associated with longer movement time 29.

Only one study assessed the on-road driving performance of persons with disabilities. Although the author did not provide specific details about the testing protocol, the outcome measures were clearly described. The testing protocol included all three dimensions of driving (motor, vision and cognition), and the use of this protocol resulted in good accuracy in predicting overall driving performance. However, the physical assessment (motor) was only partially included in the screening protocol, and its predictive power was unclear 26.

Koppa et al. also described a device used to clinically assess driving capability and to assist with adaptive equipment prescription for persons with disabilities. Their device represented an objective method to interpret muscle force capability in this specific population. In contrast to the other assessment protocols, their protocol evaluates device-related measures such as steering wheel and hand control force and provides recommendations based on these outcome measures, thus contributing to a unique area of driving assessment for those with limited physical capabilities 27.

Reger et al. 30 developed a similar, but slightly more sophisticated, approach to assess and train driving abilities in individuals with SCI. In this protocol, only the device-related measures were collected, and training sessions were performed during a 4-week period. A series of training and evaluation sessions were used to suggest the readiness of the subject for an on-road test based on specific technical recommendations, such as the force needed for brake application.

Following the same rationale of the abovementioned authors, a group of researchers developed a virtual driving simulator for SCI subjects. In addition to collecting data on driving performance using this simulator, these researchers asked participants about their overall driving experience using the driving simulator 28. Taking into account the subjects' experiences using the simulator contributed to a better understanding of the effect of being in a simulated environment during testing and its impact on cognition and the subjects' overall emotional stress.

There is a lack of evidence supporting the resumption of driving after an amputation, but many amputees resume driving after surgery. Most articles obtained from our review were dated and reflected information on driving habits and driving preferences after amputation. After screening, we found only two articles on driving assessment after lower limb amputation.

Pauley et al. 20 found that transtibial amputees demonstrated higher RT and movement time than controls when exposed to a dual task, and this result is in accordance with previous studies in non-amputee populations 34. However, the most interesting finding was that the intact limb had a similar dual-task effect to the prosthetic leg. The authors suggested that this result might be due to the reorganization of central motor pathways after amputation.

The longest RT, movement time and response time were described by Meikle et al. 35 when transtibial amputees operated the accelerator with their prosthetic foot and the brake with their left foot. Whether to alter the pedal settings and whether to use the prosthetic foot to drive are important decisions that should be made with professional help, not solely based on the driver's preference.

### Assessing the motor components of driving

Muscle strength, endurance, coordination, range of motion and balance are some of the motor abilities required to operate basic vehicular controls. These motor abilities are very specific to the driver, regardless of the presence or lack of a disability, –and to the design of the automobile. Therefore, many factors, or a combination thereof, can affect each individual outcome when driving. The task of operating a motor vehicle requires multiple coordinated skills to quickly and accurately execute movements in the upper and lower limbs 36. Although physical motor function has been described as a potential component of a driving assessment, there is little data supporting its impact on driving abilities.

Although there are recommendations regarding the assessment of range of motion and muscle strength, the choice of specific measures to assess driving may be related to other factors, such as automobile design and driver biomechanics, which also affect a driver's performance. One of the major barriers to including an assessment of motor function in a test battery is that most suitable tools remain considered as subjective measures, and as stated by Ball et al. 21, these tools rely on the administrator's judgment ability. In comparison, the use of more sophisticated and precise assessment tools is often considered as impractical in clinical settings and as too expensive in outpatient settings.

Although there are no parameters available for precise comparisons due to the characteristics of individual drivers and the variability of external factors, Shugg et al. 37 estimated that on average, for 13% of the driving time, subjects have their necks outside of the neutral range of motion. Additionally, researchers have indicated that limited neck range of motion can certainly alter driving performance 38.

It has been stated that during steering wheel movement, the upper limbs asymmetrically contribute to generate adequate torque during a bimanual task. Although these data were collected from healthy subjects using an isokinetic device, this study provides some indication of how the upper extremities may contribute to the steering movement cycle. Researchers suggest further investigation of these movement patterns 36, as these patterns are essential when managing populations with restricted upper extremity movement, such as persons with tetraplegia.

Those who rely on the upper extremities for driving may use adapted cars and/or a variety of assistive devices to help them drive. Interestingly, one study showed that considering the injury severity, age and activities of daily living performance, toilet transfer ability is the most reliable indicator of driving performance 22. Thus, for more severely impaired individuals, muscle strength tests and range of motion measures may be crucial. Physical limitations are even more pronounced among older persons with disabilities.

When comparing older drivers and older non-drivers, a study by Antin et al. 2 indicated significant differences in measures of lower extremity and upper body strength between these groups, indicating upper body strength as one of the five functional abilities to be included in a future driving assessment model. Alternatively, a modest association between poor performance in rapid-pace walking and the occurrence of at-fault crashes was found by Marottoli et al. 39. The Guidelines for Motor Vehicle Administrators describe the most comprehensive motor exam, which includes a detailed muscle strength examination 40. Studies demonstrated that those with scores for neck 40 and trunk rotation 39 and rapid pace walking below the respective cut-off values were more likely to be involved in an at-fault automobile accident 40. However, total neck rotation was not included by Wood et al. 31 in their test battery, as it was not found to be significant based on their regression model. Alternatively, knee extension and postural sway were found by these authors to be predictors of unsafe driving performance.

## DISCUSSION

The act of driving requires the integration of skills that should be performed together almost continuously to safely operate a motor vehicle. Multifactorial evaluations are being used for general and/or specific diagnoses 41. Although there is no consensus regarding the tools available, researchers appear to have a good understanding of methods to assess driving using current testing measures and devices.

The reliability and feasibility of assessment tools and protocols to be used in a clinical setting are critical factors to consider when evaluating the driving performance of individuals with lower extremity impairment. Most importantly, clinical judgment is key to making these evaluating decisions. It is also important to collect additional information about the impact of driving on a person's life, especially after a major health issue, so that the examiner can have a better idea of a given driver's expectations and behaviors. An individual's medical diagnosis alone may not always be the best determinant of his/her fitness to drive. Instead, his/her level of functional impairment often serves as a better indicator of the individual's driving ability by providing specific information on balance and muscle force, strength and coordination. Most of the recommended driving assessment protocols include the three domains of evaluation discussed above (cognitive, visual and motor). Alternatively, some protocols focused on the assessment of the visual and cognitive aspects of driving based on their role in predicting on-road performance 42-45. However, the challenge is to design a simple and cost-effective evaluation model that optimally assesses the broad spectrum of active drivers with a wide range of physical and emotional limitations.

Although not addressed in this article, visual abilities are also crucial for driving. Isler et al. 46 found a significant but weak correlation between severely restricted head movement and loss of peripheral vision. Their findings revealed that loss of head movement is typically compensated for by additional eye movements and that for those with serious neurological impairment, focusing on oncoming traffic would be extremely difficult if not impossible. Whereas the relationship between cognition-vision and driving ability is clear and can be measured using different tools, little is known about the impact of motor deficits on the ability to safely drive an automobile. This is especially important for drivers who resume driving following SCI or lower limb amputation.

When driving a car, persons with SCI, for example, may experience difficulties beyond the simple act of driving. While in a car, these individuals must manage environment-specific demands, such as vibration, to operate adapted car controls while maintaining a well-supported seating position 47,48. A similar phenomenon occurs when resuming driving after a lower limb amputation. The dilemma regarding whether it is appropriate to drive using the leg/foot prostheses, combined with the difficulties in handling the car pedals, illustrate some of the challenges faced by those with a physical impairment when preparing to drive.

As discussed by Prasad et al. in 2006 49, adjusting one's driving technique due to the need to use adaptive equipment or a prosthetic device can be more difficult than using familiar controls. Their study, which was based on a variety of disabled drivers, showed a high accident rate among those using hand controls and reported a low frequency of left foot accelerator/brake use. These findings demonstrated the need to re-train individuals who resume driving using unconventional controls.

It has been reported that age, gender (being female), having a right-side amputation and the presence of co-morbidities significantly reduce the likelihood of returning to driving among amputees 4,6,35. The use of a prosthetic device to drive should be carefully evaluated because amputation leads to the loss of proprioception, which is a necessary element for safely operating the car pedals.

Several of the studies discussed in this review used driving simulators as the means of evaluating and improving driving performance through training. Driving simulators can address the limitations of on-road tests, as they offer a variable environment, safe evaluation of challenging situations and the possibility of an objective and standardized assessment. Practice and improvement of former or new skills are critical for operating a motorized vehicle, especially for lower extremity-impaired individuals. Studies by Ku et al. 28 and by Sun et al. 50 were not included in our review because they did not address driving assessment *per se*. However, these studies demonstrated that the use of a driving simulator as a training tool for SCI patients can be beneficial to not only their confidence but also their driving performance 28,50.

The primary purpose of this review was to identify methods of assessing driving for able-bodied and lower extremity-impaired people. Although evidence showing a clear relationship between motor performance and driving outcomes is almost non-existent, researchers and clinicians continue to use measures that assess motor performance and related skills when evaluating one's ability to drive. The most evident finding was the paucity of information regarding driving assessment for lower extremity-impaired individuals. Additionally, only one reviewed study described the use of driving assessments during road tests; that study found that the use of a driving simulator plays an important role in the assessment of this population.

Based on our findings, we believe that the ideal driving evaluation instrument for individuals with lower extremity impairment should include the use of driving simulators to assess functional deficits in combination with functional tests designed to evaluate motor performance. Functional tests have the benefit of evaluating the individual's performance during the task. Because persons with SCI or lower limb amputation may exhibit significant muscle coordination compensation for performing impaired movements, the classical measures to assess range of motion and torque may not accurately represent the necessary skill needed to perform selected movements that are required during driving.

This knowledge must be shared with politicians, government agencies and lawmakers so that this information can be used to improve the process of acquiring a driver's license, as although this review may be more useful to researchers and students exploring this particular matter.

This review has outlined the current methods of assessing driving for able-bodied people and has discussed the available tools for the evaluation of those with lower extremity impairment. Very few studies provide guidance regarding the evaluation of driving skills among persons with lower extremity impairment. The need for such studies continues to grow with the growing worldwide population of older individuals with co-morbidities and/or traumatic injuries that may decrease lower extremity function and thus affect driving capability. Future studies may further assist health care practitioners in developing a comprehensive assessment protocol for lower limb-impaired individuals.

## Figures and Tables

**Table 1:** Motor evaluations in the proposed driving assessment models.

Study	Population	Motor assessment	Outcome
Ball et al., 2006	Older adults	Rapid Walk Foot tap Arm reach Head/neck rotation	Physical performance was not a significant predictor of prospective crash
Eby et al., 2007	Older adults	Rapid pace walk Arm reach Head/neck rotation Hand strength	Driving assessment battery development
Stav et al., 2008	Older drivers	Head/neck flexibility Rapid pace walk Range of motion Cervical Trunk Upper extremities Lower extremities Manual muscle test Upper/lower extremities	Rapid pace walk was the only motor test included in the suggested driving assessment model
Wood et al., 2008	Older drivers	Neck range of motion Quadriceps strength Postural sway	Knee extensor, strength and postural sway were the best driving performance predictors under the motor domain
Antin et al., 2012	Drivers vs.non-drivers	Strength/torque Upper/lower body Head-neck-torso flexibility	Physical dimension was part of a developed model
Marshall et al., 2013	Older drivers	Range of motion Manual test of motor strength Timed up and go Rapid pace walk One-leg stance	Driving assessment battery development
Ott et al., 2013	Normal cognition vs.Cognitive impairment	Rapid pace walk Strength Range of motion Neck Limbs	Rapid pace walk and range of motion were included as part of a set of tests able to predict driving performance
Woolnough et al., 2013		Rapid Pace Walk Manual Range of Motion Neck Shoulder flexion Elbow flexion Finger curl Ankleplantar flexion/dorsiflexion Manual test of strength Shoulder Adduction/abduction Wrist flexion/extension Ankle dorsiflexion/plantar Flexion	No association with crash involvement
Lacherez et al., 2014	Older drivers	Muscle strength Quadriceps Ankle dorsiflexion Hand grip Range of motion Neck	Quadriceps strength was part of a three variable model to discriminate safe from unsafe drivers

**Table 2:** Primary outcomes and tools used in the selected studies of driving assessments for lower extremity-impaired individuals.

Authors	Population (n)	Instrument	Outcomes
Koppa et al.,1978	SCI subjects(7)	Driving control measurement device	Range of motion;*
			Steering wheel force;
			Hand control force
Reger et al., 1981	Tetraplegic subjects (9)	Driving simulator	Brake force;
			Shoulder rotation;
			Elbow extension
Gouvier et al., 1989	Disabled subjects**	Battery of psychometric and performance tests	Strength;Range of motion
Lings, S., 1991	Subjects with paraparesis inferior (52)	Driving simulator	Grip strength;
			Brake force;
			Steering wheel speed;
			Reaction time
Ku et al., 2002	Able-bodied subjects (10) SCI subjects (15)	Driving simulator	Speed;
			Steering stability;
			Traffic signal violations;
			Centerline violations;
			Driving time
Meikle et al., 2006	R transtibial amputees	Set of brakes and accelerator and reaction time software	Reaction time;
			Movement time;
			Total response time;
			Pedal configuration preferences
Hoberry et al., 2010	Disabled individuals**	Driving simulator	Steering wheel torque;
			Steering wheel ability;
			Brake force;
			Reaction time
Pauley et al., 2011	Transtibial amputees (10) Healthy controls (13)	Reaction time/movement time system with foot switches	Reaction time;Movement time;Total response time

Notes:

* Based on the ability to turn the steering wheel.

** SCI was one of the most commonly reported impairments.
